# Elevated baseline potassium level within reference range is associated with worse clinical outcomes in hospitalised patients

**DOI:** 10.1038/s41598-017-02681-5

**Published:** 2017-05-25

**Authors:** Sehoon Park, Seon Ha Baek, Sung Woo Lee, Anna Lee, Ho Jun Chin, Ki Young Na, Yon Su Kim, Dong-Wan Chae, Jin Suk Han, Sejoong Kim

**Affiliations:** 1 Department of Biomedical Sciences, Seoul National University College of Medicine, Seoul, Korea; 2 Department of Internal Medicine, Hallym University Dongtan Sacred Heart Hospital, Gyeonggi-do, Korea; 3 Department of Internal Medicine, Eulji General Hospital, Seoul, Korea; 4 Department of Internal Medicine, Seoul National University Bundang Hospital, Gyeonggi-do, Korea; 5 Department of Internal Medicine, Seoul National University College of Medicine, Seoul, Korea

## Abstract

The clinical significance of elevated baseline serum potassium (K^+^) levels in hospitalised patients is rarely described. Hence, we performed a retrospective study assessing the significance of elevated K^+^ levels in a one-year admission cohort. Adult patients without hypokalaemia or end-stage renal disease were included. Adverse outcomes were all-cause mortality, hospital-acquired acute kidney injury, and events of arrhythmia. In total, 17,777 patients were included in the study cohort, and a significant difference (P < 0.001) was observed in mortality according to baseline serum K^+^ levels. The adjusted hazard ratios (HRs) and associated 95% confidence intervals (CIs) of all-cause mortality for K^+^ levels above the reference range of 3.6–4.0 mmol/L were as follows: 4.1–4.5 mmol/L, adjusted HR 1.075 (95% CI 0.981–1.180); 4.6–5.0 mmol/L, adjusted HR 1.261 (1.105–1.439); 5.1–5.5 mmol/L, adjusted HR 1.310 (1.009–1.700); >5.5 mmol/L, adjusted HR 2.119 (1.532–2.930). Moreover, the risks of in-hospital acute kidney injury and arrhythmia were higher in patients with serum K^+^ levels above 4.0 mmol/L and 5.5 mmol/L, respectively. In conclusion, increased serum K^+^ levels, including mild elevations may be related to worse prognosis. Close monitoring and prompt correction of underlying causes or hyperkalaemia itself is warranted for admitted patients.

## Introduction

Hyperkalaemia is one of the major electrolyte disturbances in medicine^1, 2^. This electrolyte imbalance is prevalent in patients with cardiovascular disease or impaired kidney function^3, 4^, and the recent use of several medications related to serum potassium (K^+^) levels has further increased its incidence^5–7^. Moreover, hyperkalaemia is associated with worse prognosis and can induce critical arrhythmia^8–12^. Hence, management of this electrolyte imbalance has been suggested in clinical guidelines and is widely practised^13–15^.

Multiple physiological mechanisms contribute to K^+^ homeostasis, and consequently, serum K^+^ levels are generally well regulated^16^. However, in hospitalised patients, there are numerous factors, including underlying comorbidities, ongoing illness, and medication use, that affect the serum K^+^ levels^17^. Therefore, identifying serum K^+^ level with clinical significance is an important issue for clinicians to decide when to start evaluation or correction of this electrolyte imbalance.

Recent studies have shown that optimal K^+^ levels are different from those known previously^18^, and suggest lower threshold levels for hyperkalaemia^9, 10, 19^. However, these studies included limited populations who had cardiovascular disease or were in critical care. Hence, whether elevated K^+^ levels, including mild elevations within reference range, are associated with prognosis of admitted patients remains unclear.

In the current study, we evaluated the clinical significance of baseline serum K^+^ levels on prognosis in a one-year cohort of patients who were admitted to general wards. We assessed the risks of mortality, acute kidney injury (AKI), as well as arrhythmia, and demonstrated that elevation of serum K^+^ levels was an independent risk factor for worse prognosis regardless of the presence of cardiovascular disease or impaired renal function.

## Results

### Study population

**Figure 1:**
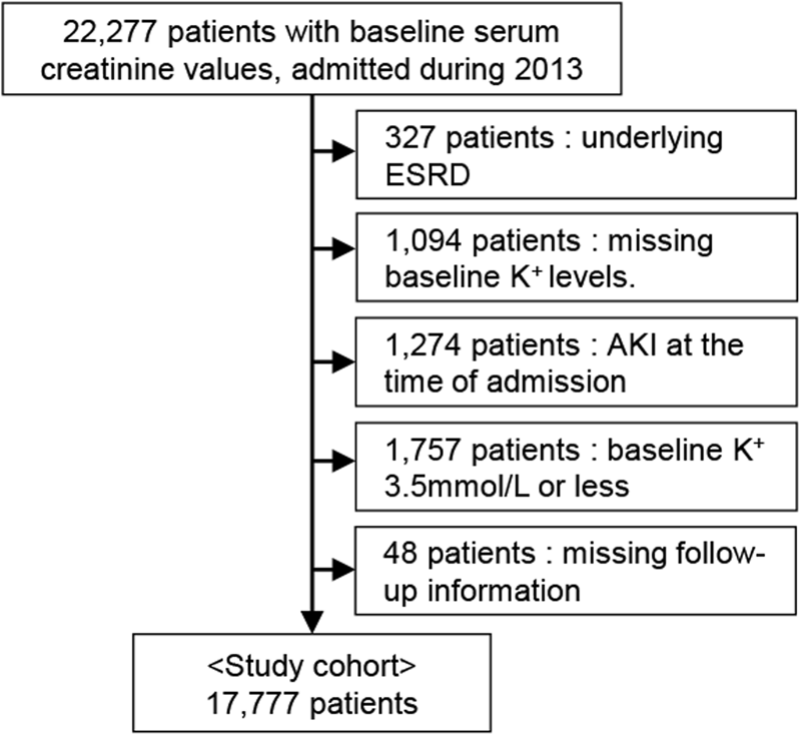
Flow diagram for the study population.

The flow diagram of the study population is shown in Fig. 1. In total, 22,277 patients were admitted whose baseline creatinine levels were available in the study year. First, we excluded patients with end-stage renal disease (ESRD) (n = 327) and those with missing baseline K^+^ values (n = 1,094). Next, patients with hypokalaemia (baseline K^+^ ≤ 3.5 mmol/L) (n = 1,757) or AKI diagnosis at the time of admission (n = 1,274) were excluded, as they were beyond the scope of the current study. Lastly, after excluding patients with missing follow-up information (n = 48), 17,777 patients were included in the final study cohort. Among these, 8,160, 7,561, 1,642, 296, and 118 patients had baseline serum K^+^ levels of 3.6–4.0 mmol/L, 4.1–4.5 mmol/L, 4.6–5.0 mmol/L, 5.1–5.5 mmol/L, and >5.5 mmol/L, respectively.

### Baseline characteristics

There were substantial differences in baseline characteristics between the study groups according to their baseline serum K^+^ levels (Table 1). Patients with higher baseline serum K^+^ levels were older (P < 0.001) and had lower body mass index (BMI) (P < 0.001). Baseline comorbidities, including cancer, ischaemic heart disease, heart failure, hypertension, and diabetes mellitus were more commonly (P < 0.001) observed in patients with higher serum K^+^ levels. As expected from the above findings, patients with supra-normal serum K^+^ levels had higher serum creatinine levels and lower estimated glomerular filtration rate (GFR) (P < 0.001). The haemoglobin and serum albumin showed similar tendencies, and decreased as baseline serum K^+^ increased. Moreover, consistent with previous findings^16, 20^, total carbon dioxide (CO_2_) levels were significantly lower in patients with elevated serum K^+^ levels (P < 0.001). Lastly, medications such as angiotensin converting enzyme inhibitors (ACE I) or angiotensin receptor blockers (ARB), beta blockers, and diuretics, regardless of diuretics’ K^+^-sparing effect, were more commonly used (P < 0.001) in patients with higher baseline K^+^ levels. In contrast, nonsteroidal anti-inflammatory drugs (NSAIDs) were more frequently used in those with lower serum K^+^

**Table 1:** Baseline characteristics according to baseline K^+^ level.

	K^+^ 3.6–4.0 (n = 8,160)	K^+^ 4.1–4.5 (n = 7,561)	K^+^ 4.6–5.0 (n = 1,642)	K^+^ 5.1–5.5 (n = 296)	K^+^ > 5.5 (n = 118)	*P value
Age (years)	58 (44–70)	60 (47–72)	65 (52–75)	69 (58–77)	68 (49–78)	<0.001
Sex (male)	3,794 (46.5)	4,462 (59.0)	1,039 (63.3)	188 (63.5)	66 (55.9)	<0.001
Body mass index (kg/m^2^)	23.7 (21.4–26.1)	23.8 (21.6–26.0)	23.7 (21.3–25.9)	22.8 (20.6–25.7)	23.1 (20.8–25.3)	<0.001
History of						
Cancer	1918 (23.5)	1928 (25.5)	456 (27.8)	77 (26.0)	35 (29.7)	<0.001
Ischaemic heart disease	165 (2.0)	253 (3.3)	52 (3.2)	19 (6.4)	2.5 (3)	<0.001
Heart failure	54 (0.7)	62 (0.8)	28 (1.7)	12 (4.1)	2 (1.7)	<0.001
Hypertension	971 (11.9)	1143 (15.1))	336 (20.5)	79 (26.7)	27 (22.9)	<0.001
Diabetes mellitus	260 (3.2)	376 (5.0)	137 (8.3)	34 (11.5)	12 (10.2)	<0.001
Laboratory results						
Serum creatinine (mg/dL)	0.6 (0.5–0.8)	0.7 (0.6–0.9)	0.8 (0.6–1.0)	0.9 (0.7–1.3)	0.9 (0.7–1.5)	<0.001
Estimated GFR (mL/min/1.73 m^2^)	74.6 (74.3–96.1)	74.5 (74.3–88.3)	74.5 (73.5–83.5)	74.3 (46.6–74.7)	73.8 (40.3–74.4)	<0.001
Albumin (g/dL)	4.0 (3.6–4.3)	4.1 (3.7–4.4)	4.0 (3.6–4.4)	3.8 (3.3–4.3)	3.9 (3.4–4.3)	<0.001
Haemoglobin (g/dL)	12.8 (11.5–14.1)	13.1(11.7–14.4)	12.9 (11.2–14.3)	12.0 (10.1–13.7)	12.1 (9.7–13.8)	<0.001
Total CO_2_ (mmol/L)	24 (22–26)	24 (23–26)	24 (22–26)	23 (20–25)	22 (19–24)	<0.001
Baseline use of						
ACE I/ARBs	421 (5.2)	591 (7.8)	193 (11.8)	54 (18.2)	19 (16.1)	<0.001
Beta blockers	339 (4.2)	465 (6.1)	144 (8.8)	40 (13.5)	9 (7.6)	<0.001
Diuretics	307 (3.8)	286 (3.8)	117 (7.1)	43 (14.5)	19 (16.1)	<0.001
**Non-K^+^-sparing diuretics	274 (3.4)	251 (3.3)	98 (6.0)	37 (12.5)	18 (15.3)	<0.001
***K^+^-sparing diuretics	58 (0.7)	70 (0.9)	35 (2.1)	15 (5.1)	5 (4.2)	<0.001
NSAIDs	863 (10.6)	633 (8.4)	153 (9.3)	19 (6.4)	6 (5.1)	<0.001

GFR, glomerular filtration rate, CO_2_, carbon dioxide, ACE I, angiotensin converting enzyme inhibitor, ARB, angiotensin receptor blocker, NSAID, non-steroidal anti-inflammatory drug.

Values were presented as n (%) for categorical variables, and median (25% to 75% interquartile) for continuous variables (all data in the table showed non-normal distribution).

*P for trend was calculated using the linear-by-linear association for categorical variables and Kruskal-Wallis test for one-way analysis of variance for continuous variables.

**Loop diuretics, thiazides, and carbonic anhydrase inhibitors.

***Spironolactone and amiloride agents. Other medications such as triamterene or eplerenone were not prescribed in the study hospital.

levels (P < 0.001).

### All-cause mortality in the study cohort

**Figure 2:**
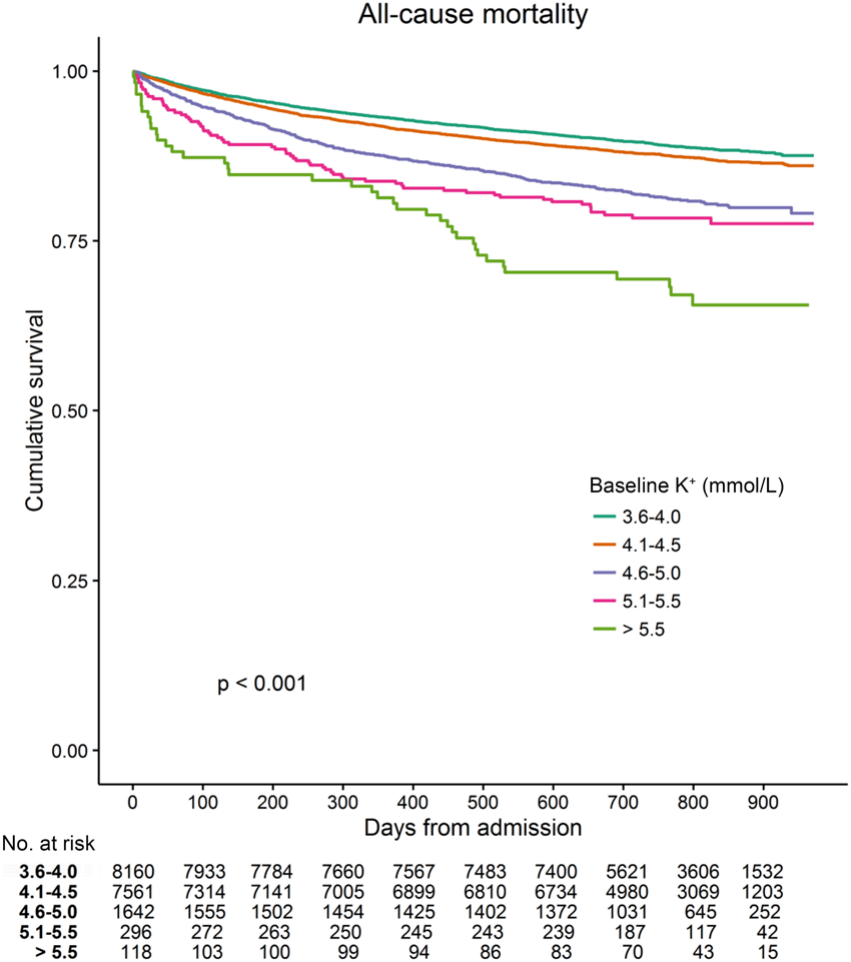
Kaplan-Meier survival curve of the study cohort according to baseline serum K^+^ levels.

**Figure 3:**
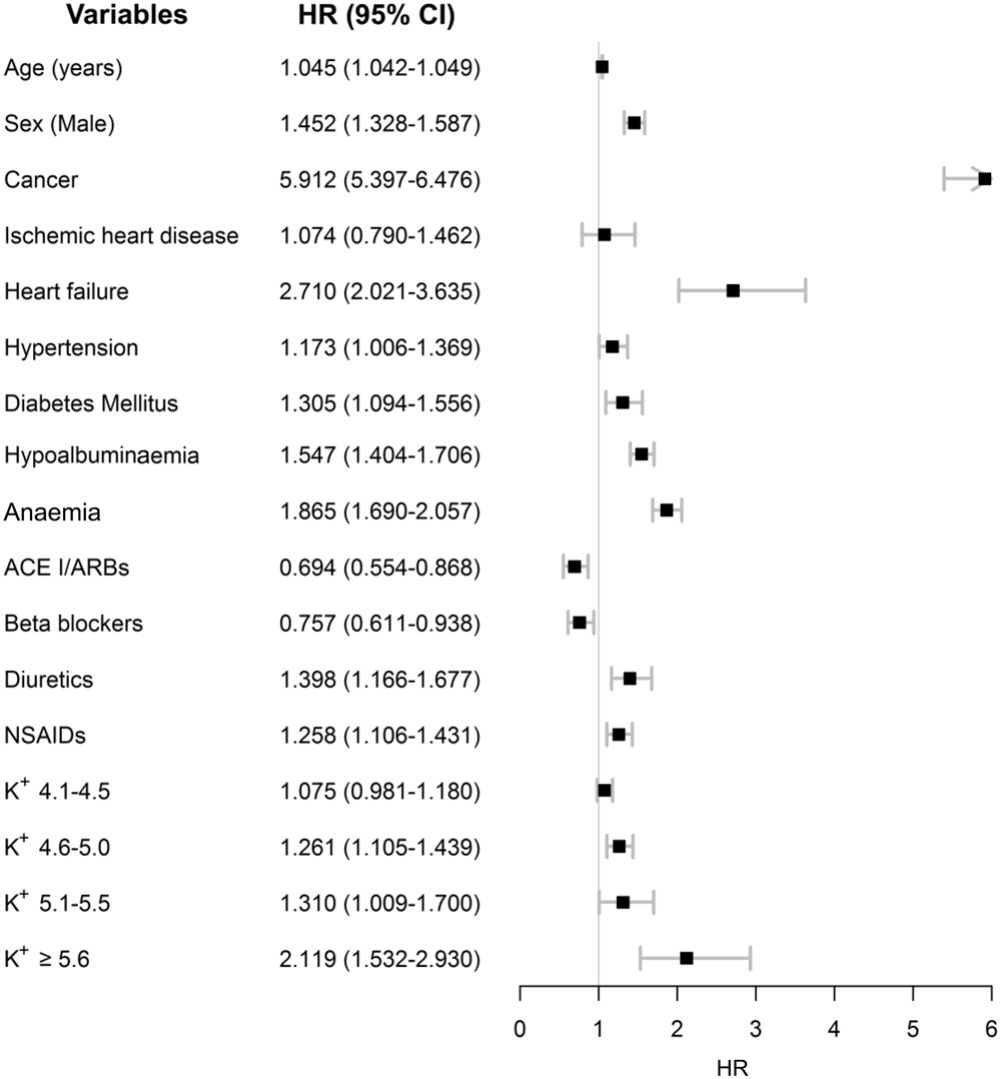
Clinical characteristics related to all-cause mortality in the study cohort. The black boxes indicate the adjusted hazard ratios (HR) for each characteristic, and the horizontal lines indicates the 95% confidence intervals (CI). The hazard ratios are adjusted for the following variables; age, sex, history of cancer, ischaemic heart disease, heart failure, hypertension, diabetes mellitus, baseline estimated GFR and total CO_2_, presence of hypoalbuminaemia (albumin level less than 3.5 g/dL), anaemia (haemoglobin level less than 11 g/dL), and baseline use of ACE I/ARBs, beta blockers, diuretics and NSAIDs. Baseline estimated GFR and total CO_2_ were included in the analysis as continuous variables (natural unit), as the laboratory findings were directly related to levels of serum K^+^.

The Kaplan-Meier survival curves demonstrating all-cause mortality for the study patients are shown in Fig. 2. Median follow-up duration of the study cohort was 2.1 (1.8–2.4) years; there were 2,278 deaths in the study cohort, of which 224 cases died within 30 days and 552 within 90 days. The five leading causes of death were cancer, respiratory disorders, cardiovascular disease, infection, and neurologic disorders, although the information was available in limited number of patients (n = 733) (see Supplementary Table S1). Interestingly, patients with higher baseline serum K^+^ levels at the time of admission had worse survival (P < 0.001). In multivariable analysis (Fig. 3), a history of cancer, which was also the most common cause of death within available information, had most prominent impact on patient survival (adjusted hazard ratio [HR] 5.912, 95% confidence interval [CI] 5.397–6.476, P < 0.001). Other previous known risk factors for worse prognosis, such as presence of anaemia (adjusted HR 1.865, 95% CI 1.690–2.057, P < 0.001) or hypoalbuminaemia (adjusted HR 1.547, 95% CI 1.404–1.706, P < 0.001) significantly correlated with all-cause mortality. A history of heart failure (adjusted HR 2.710, 95% CI 2.021–3.635, P < 0.001) and diabetes mellitus (adjusted HR 1.305, 95% CI 1.094–1.556, P = 0.003) had a strong impact on patient survival. The uses of ACE I/ARBs (adjusted HR 0.694, 95% CI 0.554–0.868, P = 0.001) and beta blockers (adjusted HR 0.757, 95% CI 0.611–0.938, P = 0.011) were protective factors for mortality, while the uses of diuretics (adjusted HR 1.398, 95% CI 1.166–1.677, P < 0.001) and NSAIDs (adjusted HR 1.258, 95% CI 1.106–1.431, P < 0.001) were associated with a higher risk of patient death. Moreover, increased risk of all-cause mortality was also observed after adjustments in patients with serum K^+^ levels 4.6–5.0 mmol/L (adjusted HR 1.261, 95% CI 1.105–1.439, P = 0.001), 5.1–5.5 mmol/L (adjusted HR 1.310, 95% CI 1.009–1.700, P = 0.043), and >5.5 mmol/L (adjusted HR 2.119, 95% CI 1.532–2.930, P < 0.001).

### 30-day and 90-day mortality and in-hospital outcomes

**Figure 4:**
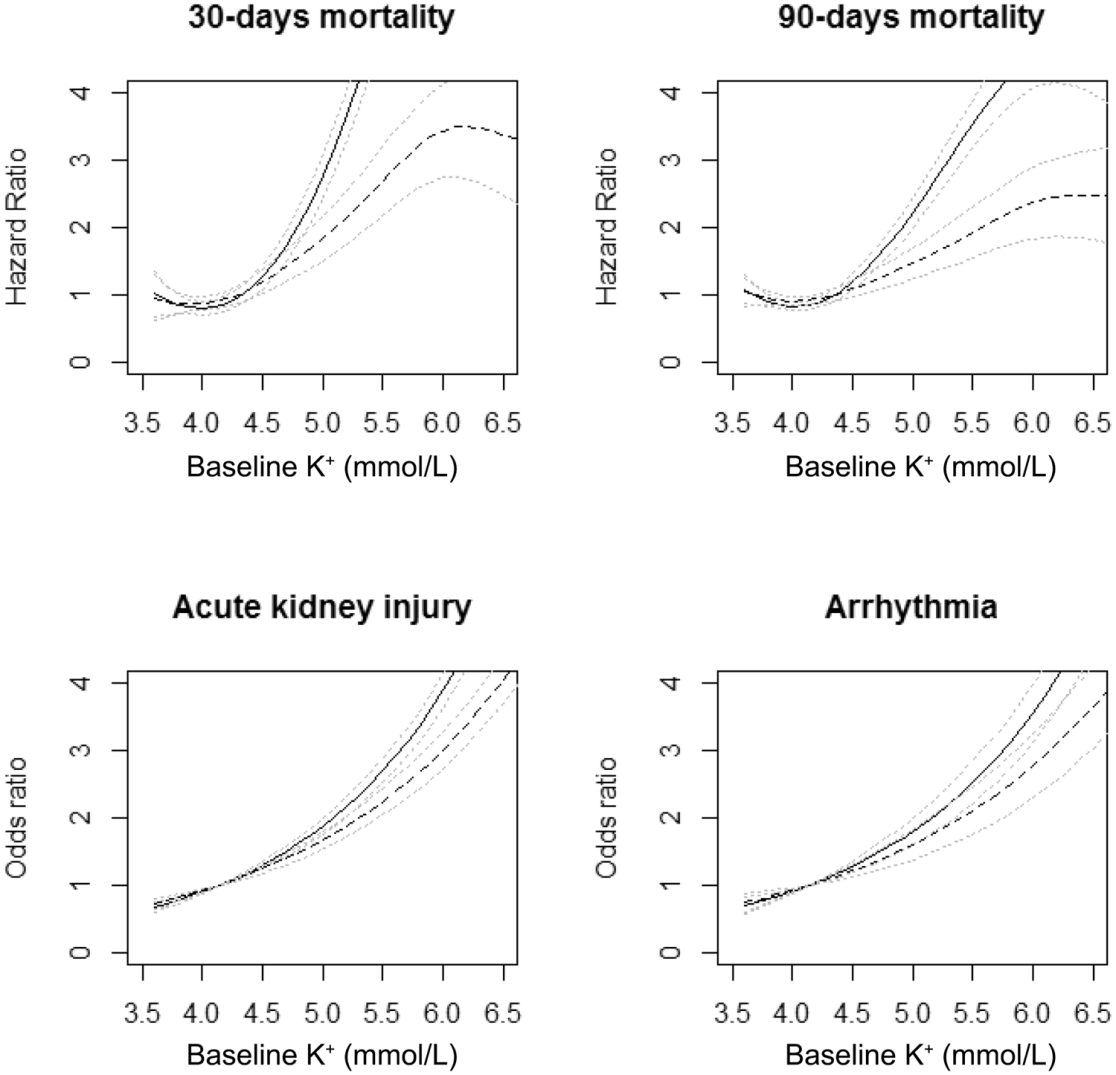
Penalized smoothing splines showing the relationship between baseline serum K^+^ levels and risks for each adverse outcome. The black, linear lines indicate the results of univariable analyses, and the black, broken lines indicate the results of multivariable analyses. The grey, dotted lines indicate the associated 95% confidence intervals. The multivariable analyses are adjusted for the following variables; age, sex, history of cancer, ischaemic heart disease, heart failure, hypertension, diabetes mellitus, baseline estimated GFR and total CO_2_, presence of hypoalbuminaemia (albumin level less than 3.5 g/dL), anaemia (haemoglobin level less than 11 g/dL), and baseline use of ACE I/ARBs, beta blockers, diuretics and NSAIDs. Baseline estimated GFR and total CO_2_ were included in the analysis as continuous variables (natural unit), as the laboratory findings were directly related to levels of serum K^+^.

We further assessed the relationship between baseline K^+^ levels and the risks of 30-day mortality, 90-day mortality, hospital-acquired AKI, and arrhythmias to evaluate short-term outcomes in detail (Table 2). Similar to the above results, the 30-day and 90-day mortalities increased in patients with serum K^+^ levels ≥4.6 mmol/L. Moreover, the risk for hospital-acquired AKI, which occurred in 895 patients, was also elevated in patients with serum K^+^ level of 4.1–4.5 mmol/L. In contrast, the risk of arrhythmia, with 260 events, was only elevated in patients with baseline serum K^+^ levels >5.5 mmol/L. Overall, the risks of mortality and adverse in-hospital outcomes showed a positive correlation with baseline K^+^ levels in both univariable and multivariable analyses (Fig. 4).

**Table 2:** Relationship between baseline serum potassium level and mortality, in-hospital acute kidney injury and in-hospital arrhythmia.

Serum K^+^ (mmol/L)	30-days mortality			90-days mortality			In-hospital acute kidney injury			In-hospital arrhythmia		
	*Adjusted HR	95% CI	P value	*Adjusted HR	95% CI	P value	*Adjusted OR	95% CI	P value	*Adjusted OR	95% CI	P value
Entire cohort (n = 17,777)												
4.1–4.5	1.260	0.926–1.716	0.142	1.159	0.957–1.405	0.131	1.235	1.053–1.450	0.010	1.147	0.869–1.514	0.332
4.6–5.0	1.563	1.037–2.356	0.033	1.358	1.044–1.767	0.023	1.668	1.329–2.093	<0.001	1.219	0.791–1.879	0.368
5.1–5.5	2.158	1.149–4.053	0.017	1.573	1.000–2.476	0.050	3.057	2.130–4.388	<0.001	1.089	0.454–2.610	0.849
>5.5	4.076	2.024–8.211	<0.001	2.758	1.605–4.740	<0.001	3.609	2.121–6.141	<0.001	4.851	2.162–10.844	<0.001
eGFR ≥ 60 (n = 16,483)												
4.1–4.5	1.327	0.958–1.839	0.089	1.189	0.968–1.460	0.099	1.157	0.974–1.374	0.096	1.252	0.934–1.678	0.133
4.6–5.0	1.681	1.082–2.612	0.021	1.377	1.030–1.842	0.031	1.598	1.241–2.056	<0.001	1.388	0.870–2.215	0.169
5.1–5.5	2.595	1.279–5.263	0.008	1.810	1.030–1.842	0.025	2.433	1.531–3.866	<0.001	1.139	0.348–3.726	0.830
>5.5	6.327	2.709–14.776	<0.001	3.718	1.894–7.298	<0.001	3.464	1.733–6.927	<0.001	7.788	3.161–19.188	<0.001
eGFR < 60 (n = 1,294)												
4.1–4.5	0.811	0.298–2.206	0.681	0.887	0.512–1.535	0.667	1.832	1.099–3.053	0.020	0.471	0.188–1.183	0.109
4.6–5.0	0.839	0.260–2.705	0.769	1.050	0.556–1.984	0.880	1.598	0.882–2.896	0.122	0.537	0.174–1.652	0.537
5.1–5.5	1.069	0.243–4.703	0.930	0.717	0.265–1.938	0.512	3.096	1.539–6.226	0.002	0.834	0.197–3.525	0.805
>5.5	2.386	0.597–9.535	0.219	1.829	0.691–4.844	0.224	2.611	1.038–6.569	0.042	1.773	0.310–10.151	0.520

HR, hazard ratio by Cox regression hazard model, OR, odds ratio by logistic regression analyses, CI, confidence interval, eGFR, estimated glomerular filtration rate (mL/min/1.73 m^2^).

The reference range of the analysis was serum K^+^ 3.6–4.0 mmol/L.

*Adjusted with the following variables; age, sex, history of cancer, ischaemic heart disease, heart failure, hypertension, diabetes mellitus, baseline estimated GFR and total CO_2_, presence of hypoalbuminaemia (albumin level less than 3.5 g/dL), anaemia (haemoglobin level less than 11 g/dL), and baseline use of ACE I/ARBs, beta blockers, diuretics and NSAIDs. Baseline estimated GFR and total CO_2_ were included in the analysis as continuous variables (natural unit), as the laboratory findings were directly related to levels of serum K^+^.

In addition, subgroup analyses were performed according to the presence of baseline renal function impairment (estimated GFR < 60 mL/min/1.73 m^2^), as this factor showed a significant interaction with patient survival (P = 0.013). Unexpectedly, the association of baseline serum K^+^ levels with patient survival was more prominent in patients without reduced estimated GFR values, and only the AKI risk showed a significant association with serum K^+^ elevation in patients with estimated GFR < 60 mL/min/1.73 m^2^.

Lastly, as the in-hospital outcomes might have affected patient survival, we further tested the above relationship in patients without events of AKI or arrhythmia during admission (Table 3). Patients with serum K^+^ levels of 5.1–5.5 mmol/L or >5.5 mmol/L still had an increased risk for 30-day mortality. Similar results were observed for 90-day mortality; however, statistical significance was not achieved in patients with baseline K^+^ levels of 5.1–5.5 mmol/L.

**Table 3:** Association of baseline serum K^+^ range and mortality in patients without events of acute kidney injury or arrhythmia.

Serum K^+^ (mmol/L)	30-days mortality			90-days mortality		
	*Adjusted HR	95% CI	P value	*Adjusted HR	95% CI	P value
AKI (-) (n = 16,882)						
4.1–4.5	1.328	0.886–1.991	0.169	1.221	0.976–1.527	0.080
4.6–5.0	1.697	0.970–2.970	0.064	1.393	1.014–1.912	0.041
5.1–5.5	2.669	1.045–6.817	0.040	1.704	0.917–3.165	0.092
>5.5	6.482	2.473–16.992	<0.001	2.877	1.391–5.950	0.004
Arrhythmia (-) (n = 17,517)						
4.1–4.5	1.244	0.909–1.701	0.173	1.146	0.945–1.391	0.165
4.6–5.0	1.458	0.953–2.231	0.082	1.311	1.004–1.712	0.047
5.1–5.5	2.303	1.225–4.330	0.010	1.615	1.026–2.543	0.038
>5.5	2.651	1.115–6.304	0.027	2.102	1.129–3.915	0.019
AKI (-) & Arrhythmia (-) (n = 16,649)						
4.1–4.5	1.318	0.880–1.976	0.181	1.214	0.971–1.518	0.090
4.6–5.0	1.590	0.898–2.814	0.111	1.358	0.987–1.869	0.060
5.1–5.5	2.650	1.037–6.772	0.042	1.691	0.910–3.142	0.096
>5.5	5.216	1.806–15.059	0.002	2.489	1.149–5.390	0.021

HR, hazard ratio by Cox regression hazard model, CI, confidence interval, AKI, acute kidney injury.

The reference range of the analysis was serum K^+^ 3.6–4.0 mmol/L.

*Adjusted with the following variables; age, sex, history of cancer, ischaemic heart disease, heart failure, hypertension, diabetes mellitus, baseline estimated GFR and total CO_2_, presence of hypoalbuminaemia (albumin level less than 3.5 g/dL), anaemia (haemoglobin level less than 11 g/dL), and baseline use of ACE I/ARBs, beta blockers, diuretics and NSAIDs. Baseline estimated GFR and total CO_2_ were included in the analysis as continuous variables (natural unit), as the laboratory findings were directly related to levels of serum K^+^.

### Characteristics associated with mild hyperkalaemia

We found that short-term mortality risks considerably increased with baseline K^+^ values of 5.1–5.5 mmol/L even in the absence of hospital-acquired AKI or arrhythmia; therefore, we performed an additional analysis to identify the clinical factors related to presence of mild baseline hyperkalaemia (Table 4). Almost all variables were significantly associated with the mild hyperkalaemia in univariable analysis, except for the history of cancer (odds ratio [OR] 1.067, 95% CI 0.821–1.387, P = 0.625) and the use of NSAIDs (OR 0.654, 95% CI 0.410–1.043, P = 0.075). In the multivariable logistic regression model, patients with K^+^ level of 5.1–5.5 were older (adjusted OR 1.015, 95% CI 1.006–1.023, P = 0.001) and more frequently male sex (adjusted OR 1.738, 95% CI 1.350–2.238, P < 0.001) compared to those with lower (3.6–5.0 mmol/L) K^+^ levels. As expected, estimated GFR (adjusted OR 0.988, 95% CI 0.983–0.994, P < 0.001) and total CO_2_ levels (adjusted OR 0.894, 95% CI 0.857–0.933, P < 0.001) significantly correlated with mild hyperkalaemia, showing that reduced kidney function and acidosis were directly linked to serum K^+^

**Table 4:** Factors associated with mild hyperkalaemia (K^+^ 5.1–5.5 mmol/L).

	Univariable analysis			*Multivariable analysis		
	OR	95% CI	P value	Adjusted OR	95% CI	P value
Age (years)	1.031	1.023–1.039	<0.001	1.015	1.006–1.023	0.001
Sex (male)	1.511	1.190–1.918	0.001	1.738	1.350–2.238	<0.001
History of						
Cancer	1.067	0.821–1.387	0.625	1.042	0.787–1.381	0.773
Ischaemic heart disease	2.465	1.535–3.959	<0.001	1.391	0.833–2.323	0.208
Heart failure	5.053	2.772–9.210	<0.001	2.019	1.046–3.898	0.036
Hypertension	2.216	1.707–2.877	<0.001	0.817	0.520–1.284	0.381
Diabetes mellitus	2.785	1.934–4.010	<0.001	1.632	1.093–2.435	0.017
Laboratory results						
Estimated GFR (mL/min/1.73/m^2^)	0.972	0.966–0.978	<0.001	0.988	0.983–0.994	<0.001
Total CO_2_ (mmol/L)	0.840	0.808–0.873	<0.001	0.894	0.857–0.933	<0.001
Hypoalbuminaemia (albumin < 3.5 g/dL)	1.790	1.384–2.316	<0.001	1.019	0.752–1.381	0.903
Anaemia (Hb < 11 g/dL)	2.812	2.209–3.578	<0.001	2.207	1.650–2.952	<0.001
Baseline use of						
ACE I/ARBs	2.992	2.215–4.042	<0.001	1.574	0.928–2.668	0.092
Beta blockers	2.706	1.926–3.800	<0.001	1.369	0.909–2.064	0.133
Diuretics	3.986	2.860–5.556	<0.001	1.557	1.015–2.387	0.043
NSAIDs	0.654	0.410–1.043	0.075	0.769	0.477–1.241	0.282

OR, odds ratio by logistic regression analyses, CI, confidence interval, GFR, glomerular filtration rate, CO_2_, carbon dioxide, Hb, haemoglobin, ACE I, angiotensin converting enzyme inhibitor, ARB, angiotensin receptor blocker, NSAID, non-steroidal anti-inflammatory drug.

Those with overt hyperkalaemia (K^+^ > 5.5 mmol/L) were not considered in this table.

*Adjusted with the following variables; age, sex, history of cancer, ischaemic heart disease, heart failure, hypertension, diabetes mellitus, baseline estimated GFR and total CO_2_, presence of hypoalbuminaemia (albumin level less than 3.5 g/dL), anaemia (haemoglobin level less than 11 g/dL), and baseline use of ACE I/ARBs, beta blockers, diuretics and NSAIDs. Baseline estimated GFR and total CO_2_ were included in the analysis as continuous variables (natural unit), as the laboratory findings were directly related to levels of serum K^+^.

levels. Underlying heart failure (adjusted OR 2.019, 95% CI 1.046–3.898, P = 0.036), presence of anaemia (adjusted OR 2.207, 95% CI 1.650–2.952, P < 0.001), history of diabetes mellitus (adjusted OR 1.632, 95% CI 1.093–2.435, P = 0.017), and the use of diuretics (adjusted OR 1.557, 95% CI 1.015–2.387, P = 0.043) were other factors that significantly associated with the presence of mild hyperkalaemia in multivariable analysis. However, history of hypertension (adjusted OR 0.817, 9% CI 0.520–1.284, P = 0.381), ischaemic heart disease (adjusted OR 1.391, 95% CI 0.833–2.323, P = 0.208), hypoalbuminaemia (adjusted OR 1.019, 95% CI 0.752–1.381, P = 0.903), and the use of ACE I/ARBs (adjusted OR 1.574, 95% CI 0.928–2.668, P = 0.092), or beta blockers (adjusted OR 1.369, 95% CI 0.909–2.064, P = 0.133) lost their statistical significance in the multivariable analysis.

## Discussion

In the current study, risks of all-cause mortality, hospital-acquired AKI, and arrhythmia increased in admitted patients with elevated baseline serum K^+^ levels. Interestingly, risks of hospital-acquired AKI and short-term mortality significantly increased in patients with mild serum K^+^ elevations: ≥4.6 mmol/L for short-term mortality and ≥4.1 mmol/L for AKI; this relationship was more prominent in patients with preserved baseline kidney function. Moreover, even in patients without events of hospital-acquired AKI or arrhythmia, the risk for short-term mortality was higher in those with mild hyperkalaemia (5.1–5.5 mmol/L).

Our study is the first on to demonstrate a significant association between mild elevations in baseline serum K^+^ levels, which were previously considered normal or without clinical significance^18^, and risk of short-term mortality and AKI in patients admitted to general wards. Few previous studies have considered the impact of elevated serum K^+^ level, with most of them focussing on the relationship between serum K^+^ levels and adverse prognosis in patients with cardiovascular or chronic kidney diseases^8, 11, 12, 21–23^. Among these studies, there were evidences showing the clinical significance of elevated serum K^+^ levels, i.e., >4.7 mmol/L or >4.0 mmol/L, for worse patient prognosis^9, 10, 19^. Our study results are consistent with those findings, further confirming that the significance of elevated serum K^+^ levels is not limited to patients with cardiovascular disease or those in critical care. Moreover, the association remained valid in patients without decreased renal function (estimated GFR < 60 mL/min/1.73 m^2^), events of arrhythmia or AKI. This further emphasizes the importance of appropriately interpreting elevated serum K^+^ levels in hospitalised patients.

The presence of hyperkalaemia is a well-known, strong risk factor for critical arrhythmia^13, 15, 24, 25^. In the current study, the risk of arrhythmia was elevated in patients with serum K^+^ levels >5.5 mmol/L, consistent with the findings of previous research^13, 21, 25^. However, arrhythmia alone cannot explain the association between elevated serum K^+^ levels and adverse outcomes, as the relationship remained to be significant in patients without prominent hyperkalaemia, even after excluding patients with arrhythmia events. Although we could not determine the reasons for the adverse impact of elevated serum K^+^ levels on patient prognosis in the current study, several mechanisms could be considered. First, patients with elevated serum K^+^ levels were at risk for hospital-acquired AKI. Hence, this electrolyte imbalance might be a marker related to worse renal function, resulting from various unstable medical conditions. As AKI in hospitalised patients is closely related to an increased risk of mortality, patients with elevated K^+^ levels would have a poorer prognosis^26^. Second, reduced urinary K^+^ excretions due to depleted volume status could be one of the major causes of baseline serum K^+^ level elevations in our study cohort^16^. This is supported by the fact that a history of heart failure, use of diuretics, and presence of anaemia, in addition to the numerous traditional risk factors for hyperkalaemia, remained significantly associated with the presence of mild hyperkalaemia in our multivariable model. Third, cellular release of K^+^ due to underlying disease should be considered as a potential mechanism. However, as we adjusted several factors related to cellular shifts of K^+^, such as use of medications^5, 6, 27^, metabolic acidosis^20^, history of diabetes mellitus^28^, and cancer^29^, this mechanism alone could not completely explain the results. Lastly, direct physiological effect of elevated K^+^ levels might exist. However, mild hyperkalaemia in patients with reduced kidney function was not significantly associated with patient mortality, both in the current study and in previous reports^30, 31^. Considering that the effect, if present, would also appear in patients with chronic kidney disease, it would be unreasonable to expect direct toxicity of mildly elevated serum K^+^ levels. Also, the two leading causes of death, cancer and respiratory disorders in the study population, implied that other medical situations could be the main cause of mortality in patients with mild K^+^ elevation, rather than direct effects of serum K^+^, although the information was available in a limited number of patients.

As mentioned above, the impact of mildly elevated serum K^+^ in those with impaired kidney function was not evident in clinical outcomes, even though a small number of patients were analysed. Although overt hyperkalaemia has been known to be correlated with worse survival in patients with reduced renal function, in the same studies, mild hyperkalaemia below 5.5 mmol/L was not associated with death in chronic kidney disease patients^30, 31^. Elevated serum K^+^ levels in many patients with reduced estimated GFR values might be the consequence of their chronically impaired kidney function, rather than results of other unstable medical conditions. Therefore, the clinical outcomes of mildly elevated serum K^+^ levels might be different in those with decreased GFR.

Our study has several limitations mainly because of its observational nature. First, the study examined a one-year cohort in a single centre, and the patient disease status and causes for admission were heterogeneous. Even after adjusting for multiple characteristics, there could be additional hidden confounders. Second, the current study could not directly identify the mechanism behind the relationship between serum K^+^ elevation and worse prognosis. Moreover, the cause of death was available in only a partial group of the study cohort. Third, our study did not assess the effects of potassium supplements or K^+^ lowering therapy; therefore, whether modifying serum K^+^ levels or treating underlying diseases is important for improved patient prognosis could not be confirmed in this study. Lastly, the patient severity, considering the high mortality reported in our cohort, might not be the same in other clinical settings.

In conclusion, even mild elevations in serum K^+^ within the reference range were related to increased risks of adverse outcomes. The elevations in serum K^+^ level associated with an elevated risk of short-term mortality were observed to be lower than previously thought and even mild hyperkalaemia was an independent risk factor for worse patient survival, regardless of kidney injury or arrhythmia. Therefore, clinicians should closely evaluate the causes and monitor potential adverse outcomes of elevated serum K^+^ levels in hospitalised patients, especially in those with serum K^+^ elevation despite their preserved renal function.

## Patients and Methods

### Ethics statement

This study was approved by the institutional review board (IRB) of Seoul National University Bundang Hospital (IRB number: B-1511-322-114). This study was conducted in accordance with the principles of the Declaration of Helsinki. Informed consent was waived as the study was an observational cohort study without medical interventions.

### Study cohort

The study cohort consisted of data from the first admission of adults in 2013 at a tertiary teaching hospital in Korea. The inclusion criteria were: 1) age ≥18 years and 2) patients with assessment of their renal functions based on serum creatinine measurements during admission. The exclusion criteria were: 1) ESRD patients receiving renal replacement therapies, 2) missing data on baseline serum K^+^ level, 3) confirmed AKI at the time of baseline evaluation as community-acquired kidney injuries were not within the scope of this study, 4) missing follow-up information, and 5) baseline serum K^+^ levels ≤3.5 mmol/L, as we did not intend to evaluate the effects of hypokalaemia. Finally, the study cohort was divided into five groups according to baseline K^+^ levels: 3.6–4.0 mmol/L, 4.1–4.5 mmol/L, 4.6–5.0 mmol/L, 5.1–5.5 mmol/L, and >5.5 mmol/L, respectively.

### Data collection

We collected the following demographic, laboratory, and clinical information of the patients by an electronic health record (EHR) review. At first, the age, sex, and baseline BMI of the patients were recorded. Medical conditions related to serum K^+^ levels were noted including; history of cancer, ischaemic heart disease, heart failure, diabetes mellitus, and hypertension identified based on an ICD-10 diagnostic code review and the use of relative medications. Laboratory examination results, including serum K^+^, creatinine, serum albumin, haemoglobin, and total CO_2_ levels were reviewed, and the first measured level during index admission was defined as the baseline value. Laboratory values suspected to be the results of haemolysis or inadequate sampling were reported by our laboratory medicine department and excluded in the current study to exclude the effects of pseudo-hyperkalaemia. The baseline estimated GFR values were calculated using the Modification of Diet in Renal Disease (MDRD) equation^32^, and a cut-off level of estimated GFR 60 mL/min/1.73 m^2^ was used to define patients with impaired kidney function. Additionally, history of medication use was collected, including the use of ACE I/ARBs, beta blockers, diuretics, and NSAIDs, as these medications are well-known to affect the serum K^+^ level^5–7, 27, 33^. Diuretic agents were further classified according to presence of K^+^-sparing effect; loop diuretics, thiazides, and carbonic anhydrase inhibitors were classified as non-K^+^-sparing agents; and spironolactone and amiloride agents were classified as K^+^-sparing diuretics. Other known diuretic medications with K^+^-sparing effect, such as eplerenone or triamterene, were not prescribed in the study hospital.

### Outcome measurement

The main outcome of the current study was all-cause mortality. All mortality cases were first identified by the EHR review. Further, to include death events that occurred outside the study hospital, the death registry maintained by Statistics Korea (www.kostat.go.kr) which, tracking the date of deaths of all Korean people, was reviewed after approval by the government organisation. Short-term mortality was evaluated by further stratifying the events of all-cause mortality into 30-day and 90-day mortalities. We identified all causes of death which occurred in the study hospital; however, we could not clarify causes of death in the entire cohort as the complete information was unavailable in the national database we used for the current study.

In addition, hospital-acquired AKI and events of arrhythmia were included. Hospital-acquired AKI was defined as ≥1.5-fold elevation of serum creatinine levels from baseline during admission^34^. Any event of arrhythmia during hospitalisation was identified through the EHR review.

### Missing data

There were several characteristics with missing values in the current study cohort. The variable with the most frequent missing record was baseline BMI, which was lacking in 1645/17,777 (9.3%) patients; therefore, it was excluded in the multivariable analyses. Other laboratory values had the following rates of missing data: serum albumin (266/17,777, 1.5%), haemoglobin (211/17,777, 1.2%), and total CO_2_ levels (71/17,777, 0.4%). Other characteristics, including age, sex, and medical history or medication use had no missing information. As we used the complete case analysis method, 17,402 patients were included in the multivariable analyses.

### Statistical analysis

Data were presented as frequencies and percentages for categorical variables. Continuous variables were expressed as median scores (interquartile ranges), as the results of the Shapiro-Wilk normality test revealed that all continuous variables in the study showed non-normality. For baseline characteristics, we used linear-by-linear association for categorical variables and Kruskal-Wallis one-way analysis of variance for continuous variables, to calculate the P values for trends according to subgroups divided by baseline K^+^ levels. All-cause mortality according to baseline K^+^ levels was evaluated using the Kaplan-Meier survival curve with log-rank test. For multivariable analyses, survival outcomes were assessed using the Cox proportional hazard model, with adjustments of the following clinically relevant variables: age, sex, histories of cancer, ischaemic heart disease, heart failure, hypertension, diabetes mellitus, baseline estimated GFR and total CO_2_ levels, presence of hypoalbuminaemia (serum albumin level <3.5 g/dL) and anaemia (haemoglobin level <11 g/dL), and baseline use of ACE I/ARBs, beta blockers, diuretics, and NSAIDs. Multivariable logistic regression analyses were performed to evaluate whether baseline K^+^ levels were related to the events of hospital-acquired AKI or arrhythmia, which were adjusted with the same variables as in the Cox regression analyses. The relationships between serum K^+^ levels and risk of adverse outcomes were plotted using the penalized smoothing spline method, using the ‘pspline’ package in R. All statistical analyses were performed using the R package version 3.2.5. Two-sided P values with a statistical significance level <0.05 were used.

## Electronic supplementary material

Supplementary Table S1
