# Supplementary material for: Elevated baseline potassium level within reference range is associated with worse clinical outcomes in hospitalised patients
Source: Sci Rep. 2017 May 25;7:2402. doi: 10.1038/s41598-017-02681-5 (PMC5445083; doi:10.1038/s41598-017-02681-5)
Supplement: Supplementary file 1 — Supplementary Table S1 [file 41598_2017_2681_MOESM1_ESM.doc]

**Elevated baseline potassium level within reference range is associated with worse clinical outcomes in hospitalised patients**

Sehoon Park^1^, Seon Ha Baek^2^, Sung Woo Lee^3^, Anna Lee^4^, Ho Jun Chin^4,5^, Ki Young Na^4,5^, Yon Su Kim^1,5^, Dong-Wan Chae^4,5^, Jin Suk Han^5^, and *Sejoong Kim^4,5^

**Author affiliations**

^1^Department of Biomedical Sciences, Seoul National University College of Medicine, Seoul, Korea

^2^Department of Internal Medicine, Hallym University Dongtan Sacred Heart Hospital, Gyeonggi-do, Korea

^3^Department of Internal Medicine, Eulji General Hospital, Seoul, Korea

^4^Department of Internal Medicine, Seoul National University Bundang Hospital, Gyeonggi-do, Korea

^5^Department of Internal Medicine, Seoul National University College of Medicine, Seoul, Korea

**Supplementary Table S1. Available cause of death in the study population.**

|  | Total cohort | K^+^ 3.6-4.0 | K^+^ 4.1-4.5 | K^+^ 4.6-5.0 | K^+^ 5.1-5.5 | K^+^ > 5.5 |
| --- | --- | --- | --- | --- | --- | --- |
| Number of total mortality cases | 2,278 | 909 | 955 | 311 | 64 | 39 |
| Number of mortality cases  with available cause of death | 733 | 284 | 306 | 108 | 23 | 12 |
| Causes of death |  |  |  |  |  |  |
| Cancer | 390 (53.1) | 157 (55.3) | 162 (52.9) | 64 (59.3) | 5 (21.7) | 2 (16.7) |
| Respiratory disorders | 109 (14.9) | 37 (13.0) | 51 (16.7) | 9 (8.3) | 7 (30.4) | 5 (41.7) |
| Cardiovascular disease | 61 (8.3) | 23 (8.1) | 24 (7.8) | 11 (10.2) | 3 (13.0) | 0 (0.0) |
| Infection | 47 (6.4) | 19 (6.7) | 18 (5.9) | 7 (6.5) | 3 (13.0) | 0 (0.0) |
| Neurologic disorders | 45 (6.1) | 17 (6.0) | 20 (6.5) | 5 (4.6) | 3 (13.0) | 0 (0.0) |
| Liver or pancreatobiliary disease | 32 (4.4) | 13 (4.6) | 13 (4.2) | 4 (3.7) | 2 (8.7) | 0 (0.0) |
| Bleeding or trauma | 17 (2.3) | 6 (2.1) | 7 (2.3) | 3 (2.8) | 0 (0.0) | 1 (8.3) |
| Renal failure | 8 (1.1) | 1 (0.4) | 3 (1.0) | 2 (1.9) | 0 (0.0) | 2 (16.7) |
| Gastrointestinal tract disorders | 4 (0.5) | 1 (0.4) | 2 (0.7) | 1 (0.9) | 0 (0.0) | 0 (0.0) |
| Miscellaneous | 20 (2.7) | 10 (3.5) | 6 (2.0) | 2 (1.9) | 0 (0.0) | 2 (16.7) |

The numbers of patients with according cause of death were presented as n (%).
